# Effects of disease, antibiotic treatment and recovery trajectory on the microbiome of farmed seabass (*Dicentrarchus labrax*)

**DOI:** 10.1038/s41598-019-55314-4

**Published:** 2019-12-12

**Authors:** Daniela Rosado, Raquel Xavier, Ricardo Severino, Fernando Tavares, Jo Cable, Marcos Pérez-Losada

**Affiliations:** 1 CIBIO-InBIO, Centro de Investigação em Biodiversidade e Recursos Genéticos, Universidade do Porto, Campus Agrário de Vairão, 4485-661 Vairão, Portugal; 2 Piscicultura Vale da Lama, Sapal do Vale da Lama, Odiáxere, 8600-258 Lagos, Portugal; 3 Faculdade de Ciências, Departmento de Biologia, Universidade do Porto, 4169-007 Porto, Portugal; 4 School of Biosciences, Cardiff University, Cardiff, CF10 3AX UK; 5 Computational Biology Institute, Department of Epidemiology and Biostatistics, Milken Institute School of Public Health, George Washington University, Washington DC, 20052 USA

**Keywords:** Metagenomics, Microbiome

## Abstract

The mucosal surfaces of fish harbour microbial communities that can act as the first-line of defense against pathogens. Infectious diseases are one of the main constraints to aquaculture growth leading to huge economic losses. Despite their negative impacts on microbial diversity and overall fish health, antibiotics are still the method of choice to treat many such diseases. Here, we use 16 rRNA V4 metataxonomics to study over a 6 week period the dynamics of the gill and skin microbiomes of farmed seabass before, during and after a natural disease outbreak and subsequent antibiotic treatment with oxytetracycline. *Photobacterium damselae* was identified as the most probable causative agent of disease. Both infection and antibiotic treatment caused significant, although asymmetrical, changes in the microbiome composition of the gills and skin. The most dramatic changes in microbial taxonomic abundance occurred between healthy and diseased fish. Disease led to a decrease in the bacterial core diversity in the skin, whereas in the gills there was both an increase and a shift in core diversity. Oxytetracycline caused a decrease in core diversity in the gill and an increase in the skin. Severe loss of core diversity in fish mucosae demonstrates the disruptive impact of disease and antibiotic treatment on the microbial communities of healthy fish.

## Introduction

Mucosal surfaces of animals harbour microbial communities (i.e., microbiomes), which can act as the first-line of defense against pathogens, either through competition or production of antibiotic compounds^1–3^. Furthermore, microbiomes are thought to have evolved to optimize the immune response of each organ and promote homeostasis^3,4^. Usually a diverse microbiome is associated with healthy phenotypes, but disruptions to this equilibrium can lead to an increase in abundance of opportunistic pathogens and disease susceptibility^5,6^.

Many factors can shape the composition of the fish microbiomes, including host species^7^, stress^8,9^, diet^10^, water quality^11^, host physiology^12,13^ and infection^6,14,15^. Importantly, healthy mucosal surfaces, such as the skin and gills, are naturally colonized by pathogens^16–18^ from the surrounding waters that can integrate into the host’s microbial community^19,20^. A shift in the abundance of such pathogens on the fish mucosae can lead to microbial imbalance (i.e. dysbiosis) and disease^21^, which is usually accompanied by a reduction in bacterial diversity^6,14,22^.

Stress imposed by fish farming conditions can also result in changes in microbiome composition that may lead to an increase in disease susceptibility^8^. As infectious disease is one of the main constraints to aquaculture growth and profitability, it is crucial to have a better understanding of the host-symbiont-pathogen nexus. The European seabass *Dicentrarchus labrax* is one of the main farmed fish species in southern Europe, totaling 103.476 tons in landings (10% of global aquaculture production) between 2002 and 2011^23^. This important food resource is susceptible to several bacterial pathogens: *Photobacterium damselae*, which causes photobacteriosis, *Vibrio* spp. causing vibriosis, and *Tenacibaculum maritimum* causing tenacibaculosis, just to name a few^24^. All of these pathogens can induce bacterial septicemia resulting in high mortalities in fish farms^25–27^. *Photobacterium damselae* in particular, is increasingly being reported as the main etiologic agent affecting fish farms worldwide and has been also described in molluscs, crustaceans and mammals^28–34^. Control of photobacteriosis in fish farms is challenging, and mortality can reach 60–80% rates in farmed European seabass^35,36^. Although vaccination is available, immunization is still not fully effective^36,37^, and in many cases antibiotic treatment remains the preferred option to control such pathogens (e.g. Oxytetracycline^32,38^).

Most commonly, the impact of antibiotic use on fish health is assessed through toxicological studies^39,40^. The few studies that have investigated the effects of antibiotics on the microbiome of fish have focused on gut dysbiosis^41–46^. Not surprisingly, a decrease in microbial diversity was detected^42,46^, along with an increased susceptibility to secondary infection, reduced host growth^41,44^ and higher mortality^43^. Importantly, these studies also reported bacterial pathogens acquiring resistance after antibiotic treatment, suggesting that farmed fish microbiomes could become reservoirs for antibiotic resistant genes^42–44^. In fact, several studies showed an increase in resistance to tetracycline and streptomycin antibiotics in strains of *P. damselae* sampled from both wild and farmed fish hosts^35,38,47,48^.

In the present study, we characterized the dynamics of the gill and skin microbiomes of the seabass *Dicentrarchus labrax* before, during and after a disease outbreak potentially caused by *Photobacterium damselae*, and subsequent antibiotic treatment with oxytetracycline^49^. We describe the dysbiosis caused by disease and antibiotic treatment in microbial diversity of both mucosae over 3 weeks. Towards this aim, we used 16S rRNA high-throughput sequencing (metataxonomics) and amplicon sequence variance analysis to examine changes in both alpha- and beta-diversity, as well as differences in taxa proportion over time.

## Results

**Figure 1:**
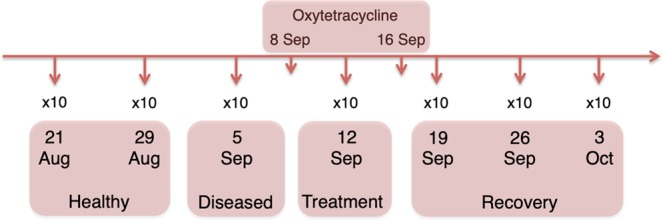
Schematic illustration of the experimental design and health status of each sampling point. Ten fish were sampled for gill and skin microbial communities at each sampling point, totaling 70 fish sampled in this experiment.

Approximately, a total of 3.6 million raw reads were generated, while the number of sequences per sample ranged from 2,354 to 50,564 (Supplementary Table S1). A total of 6,485 unique ASVs were detected, but after normalization and depletion of Archaea and Algae ASVs, a total of 3,827 ASVs (1,560,279 sequences) and 3,741 ASVs (1,904,115 sequences) were analyzed for the gill and skin microbiomes, respectively (Supplementary Table S1). Taxa showing a mean proportion ≥4% in any state were considered the most abundant taxa. Analyses of alpha- and beta-diversity showed no significant differences (P > 0.05; data not shown) between samples from the two healthy time points as well as between the samples from the three recovery time points (Fig. 1) for both gill and skin microbiomes. Therefore, in all our subsequent analyses, samples from Aug 21 (Healthy 1) and Aug 29 (Healthy 2) were combined into the “healthy” state; and samples from Sep 19 (Recovery 1), Sep 26 (Recovery 2) and Oct 3 (Recovery 3) were combined into the “recovery” state in order to increase sample size (Fig. 1).

### Gill bacterial composition and diversity

**Figure 2:**
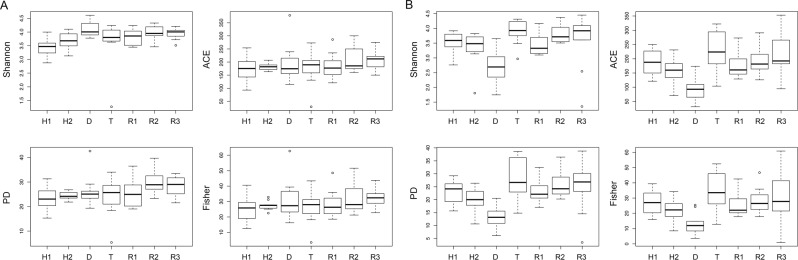
Mean values and standard deviations of Shannon, Faith’s phylogenetic (PD), ACE and Fisher alpha-diversity estimates plotted for the gill (**A**) and skin (**B**) microbiomes of *Dicentrarchus labrax* (seabass) during the four different states. H1 – Healthy 1; H2 – Healthy 2; D – Diseased; T – Treatment; R1 – Recovery 1; R2 – Recovery 2; R3 – Recovery 3.

**Figure 3:**
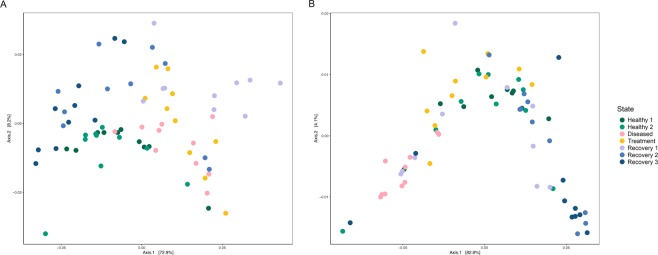
PCoA plot computed with weighted Unifrac distance for gill (**A**) and skin (**B**). Each dot represents a microbiome sample and is coloured by sampling point.

No significant differences were detected in alpha-diversity across all states (RRPP, P > 0.5), with the exception of the Shannon index (RRPP, P = 0.04) (Table 1, Fig. 2A). There were, however, significant differences between healthy and recovery states for all alpha-diversity indices (RRPP, P ≤ 0.03; Table 1). Beta-diversity also varied greatly between states (PCoA, Fig. 3A), with significant differences both in overall and pairwise comparisons in almost all the tests (Adonis, P < 0.05; Table 1).

**Table 1:** Microbial diversity and mean relative proportions of dominant taxa in the gill and skin of the seabass *Dicentrarchus labrax* (seabass) across all samples and between the four different states (H = Healthy; D = Diseased; T = Treatment; R = Recovery).

	GILL					SKIN				
	All	H vs D	D vs T	T vs R	H vs R	All	H vs D	D vs T	T vs R	H vs R
Alpha-diversity										
Shannon	**3** **(0.04)**	**19.4 (0.001)**	**2.6 (0.04)**	2.9 (0.1)	**19.7 (0.001)**	**5.1** **(0.001)**	**14.1 (0.002)**	**28.2 (0.002)**	1.6 (0.2)	1.2 (0.3)
ACE	0.9 (0.47)	0.9 (0.4)	0.4 (0.6)	1.7 (0.2)	4.1 (0.05)	**10.7** **(8**^**−6**^**)**	**18.2 (0.002)**	**24.6 (0.002)**	2 (0.2)	1.8 (0.2)
PD	2 (0.06)	1.6 (0.2)	0.5 (0.6)	3.1 (0.1)	**8.7 (0.01)**	**6** **(0.0001)**	**21.3 (0.002)**	**27.7 (0.001)**	1.4 (0.3)	3.1 (0.1)
Fisher	1.2 (0.3)	2.2 (0.2)	0.9 (0.4)	2.3 (0.1)	**4.9 (0.03)**	**4.2** **(0.002)**	**16.1 (0.003)**	**21.8 (0.002)**	2 (0.2)	1.2 (0.3)
Beta-diversity										
Uni Un	**0.2** **(9**^**−5**^**)**	**0.1** **(2**^**−4**^**)**	**0.1** **(3**^**−4**^**)**	**0.1** **(4**^**−4**^**)**	**0.1** **(9**^**−5**^**)**	**0.2** **(9**^**−5**^**)**	**0.1** **(9**^**−5**^**)**	**0.1** **(2**^**−4**^**)**	**0.04** **(4**^**−4**^**)**	**0.1** **(9**^**−5**^**)**
Uni Weigh	**0.5** **(9**^**−5**^**)**	**0.3** **(4-4)**	1.7 (0.2)	**0.1 (0.04)**	**0.1 (0.03)**	**0.4** **(9**^**−5**^**)**	**0.4** **(2**^**−4**^**)**	**0.5** **(2**^**−4**^**)**	**0.1 (0.01)**	**0.1 (0.04)**
Bray C	**0.4** **(9**^**−5**^**)**	**0.2** **(9-5)**	**0.2** **(9**^**−5**^**)**	**0.1 (0.002)**	**0.2** **(9**^**−5**^**)**	**0.3** **(9**^**−5**^**)**	**0.2** **(2**^**−4**^**)**	**0.3** **(9**^**−5**^**)**	**0.07 (0.003)**	**0.1** **(9**^**−5**^**)**
Phylum										
Bacteroidetes	**5** **(0.003)**	**11.9 (0.002)**	0.5 (0.5)	**4.7 (0.04)**	2 (0.2)	**10** **(2**^**−5**^**)**	**24.9** **(3**^**−5**^**)**	**23.6 (0.0001)**	2.2 (0.2)	1.1 (0.3)
Proteobacteria	**2.9** **(0.04)**	**5.5** **(0.03)**	0.3 (0.6)	**4.2 (0.04)**	0.1 (0.7)	**10.4** **(1**^**−5**^**)**	**21.5** **(8**^**−5**^**)**	**29.7** **(4**^**−5**^**)**	0.3 (0.6)	1.3 (0.3)
Verrucomicrobia	**4.9** **(0.004)**	3.8 (0.1)	1.2 (0.3)	**5.3 (0.03)**	**6** **(0.02)**	**3.8** **(0.01)**	**9.1 (0.005)**	**10.2 (0.005)**	0.3 (0.6)	0.8 (0.4)
Genus										
*NS3a marine group*	**5.9** **(0.001)**	**11** **(0.003)**	**6.8 (0.02)**	1.9 (0.2)	**4.5 (0.04)**	**8** **(0.0001)**	**11.5 (0.002)**	**16.7 (0.001)**	0.02 (0.9)	**5.2** **(0.03)**
*Photobacterium*	**3.1** **(0.03)**	3.3 (0.1)	1.7 (0.2)	**4.4 (0.04)**	3.8 (0.1)	—	—	—	—	—
*Polaribacter 4*	**7.5** **(0.0002)**	**12.8 (0.001)**	**5.5 (0.03)**	**7.6 (0.01)**	3.5 (0.1)	**9.1** **(4**^**−5**^**)**	**21.6** **(7**^**−5**^**)**	0.7 (0.4)	**6.1 (0.02)**	3.3 (0.1)
*Polynucleobacter*	**8.7** **(6**^**−5**^**)**	**75.8** **(2**^**−9**^**)**	2.1 (0.2)	4 (0.05)	**13 (0.001)**	—	—	—	—	—
*Pseudoalteromonas*	—	—	—	—	—	**7.4** **(0.0002)**	**12.4 (0.002)**	**6.2** **(0.02)**	**8.6 (0.01)**	2.6 (0.1)
*Pseudomonas*	**4** **(0.01)**	**7.4** **(0.01)**	0.2 (0.6)	2 (0.2)	**29.1** **(2**^**−6**^**)**	**12.6** **(1**^**−6**^**)**	**18.6 (0.0002)**	**56.8** **(6**^**−7**^**)**	1.5 (0.2)	0.9 (0.3)
*Rubritalea*	**4.6** **(0.01)**	**4.4** **(0.04)**	0.3 (0.6)	3.6 (0.1)	**6** **(0.02)**	—	—	—	—	—
*Stenotrophomonas*	**9.2** **(4**^**−5**^**)**	**8.5** **(0.01)**	0.1 (0.8)	1.3 (0.3)	**30.3** **(1**^**−6**^**)**	**3.5** **(0.02)**	**7.6 (0.01)**	**8.8** **(0.01)**	0.2 (0.7)	0.3 (0.6)

For each test we report relevant F (alpha-diversity indices and taxa proportions) or R^2^ (beta-diversity indices) statistic and significance (p). Significant associations are indicated in bold.

*Bacteroidetes, Proteobacteria* and *Verrucomicrobia* were the most abundant phyla retrieved from the seabass gill microbiome across states, accounting for 83% to 93% of the sequences altogether (Table 2). The most abundant genera were NS3a marine group, *Polaribacter 4*, *Pseudomonas* and *Rubritalea*, which were present in all four states (Table 2, Supplementary Fig. S1A). Other relatively abundant genera were: (a) *Polynucleobacter*, which accounted for 4–7% of the sequences in the diseased, treatment and recovery states, but only 0.2% in the healthy state; (b) *Stenotrophomonas*, represented by 5% of the sequences in the healthy state, and 2–3% in the remainder states; and (c) *Photobacterium*, which accounted for 5% of the sequences in the diseased state but ≤1 in all other states (Table 2, Supplementary Fig. S1A).

**Table 2:** Relative proportions of sequences and ASVs belonging to the most abundant (≥4%) phyla and genera in the gill and skin microbiomes of the seabass *Dicentrarchus labrax* in healthy, diseased, treatment and recovery states.

		Sequences (%)				ASVs (%)			
		Healthy	Diseased	Treatment	Recovery	Healthy	Diseased	Treatment	Recovery
GILL	**Phylum**								
	*Bacteroidetes*	30	21	19	26	22	21	21	23
	*Proteobacteria*	51	58	59	52	39	40	42	38
	*Verrucomicrobia*	12	7	5	8	2	2	2	2
	**Genus**								
	*NS3a marine group*	8	5	8	10	0.3	0.4	0.4	0.3
	*Photobacterium*	0.2	5	0.1	1	0.2	1	0.4	0.4
	*Polaribacter 4*	11	7	4	8	0.4	0.4	0.2	0.2
	*Polynucleobacter*	0.2	4	7	4	1	1	0.5	0.3
	*Pseudomonas*	15	9	9	6	1	1	1	1
	*Rubritalea*	10	5	4	6	0.2	1	0.1	0.2
	*Stenotrophomonas*	5	3	2	2	0.4	0.3	0.2	0.2
	TOTAL	2490	17743	25552	23076	1439	978	837	2171
SKIN	**Phylum**								
	Bacteroidetes	34	19	32	36	24	24	23	25
	Proteobacteria	54	72	52	50	39	44	36	38
	Verrucomicrobia	5	1	3	4	2	2	2	2
	**Genus**								
	*NS3a marine group*	9	6	11	11	1	1	1	1
	*Polaribacter 4*	12	5	6	9	0.5	1	0.3	1
	*Pseudoalteromonas*	0.1	1	5	1	0.1	0.4	0.2	0.1
	*Pseudomonas*	25	45	16	22	2	7	1	2
	*Stenotrophomonas*	8	12	6	8	1	2	0.3	1
	TOTAL	29110	27438	28598	27453	1433	530	1180	2160

Total number of sequences and ASVs are absolute values comprising all samples of a given group.

**Figure 4:**
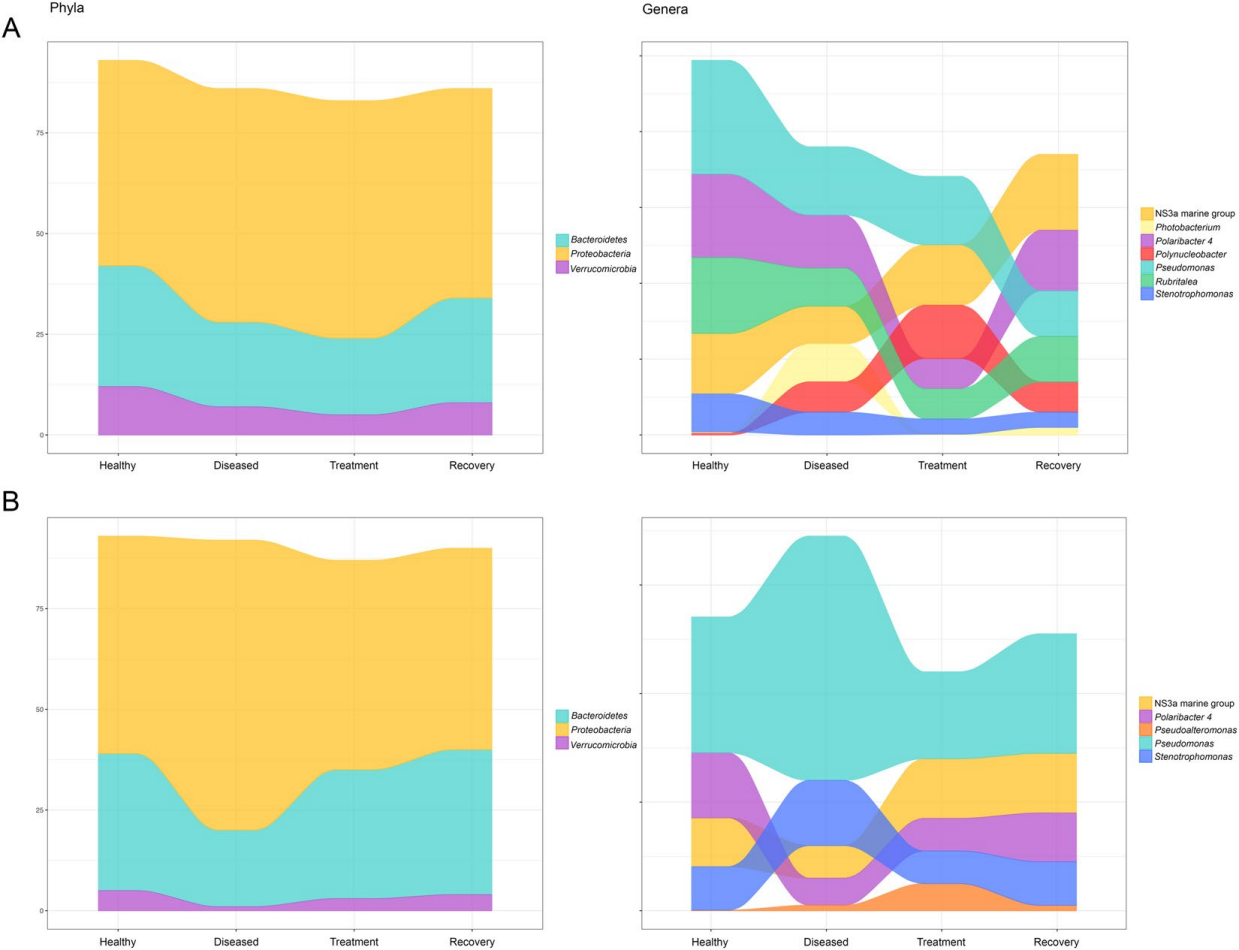
Alluvial plots of relative frequency of most abundant (>4%) taxa recovered from the gill (**A**) and skin (**B**) of the seabass for healthy, diseased, treatment and recovery states.

Taxa mean proportions varied between states: (i) in healthy versus diseased states 7 taxa increased and 3 decreased; (ii) in diseased versus treatment states 3 taxa increased, 2 decreased and 6 remained almost constant; and (iii) finally, in treatment versus recovery states 3 taxa decreased, 6 increased and 1 remained constant (Table 2, Fig. 4A). The 3 most abundant bacterial phyla and the 8 most abundant genera all varied significantly (P ≤ 0.04) in their mean proportions across the four studied states (Fig. 4A, Table 1). In addition, 9 of these taxa varied significantly (P ≤ 0.04) between healthy and disease states, whereas 5 varied significantly (P ≤ 0.04) between treatment and recovery states (Table 1). Only 2 genera varied significantly (P ≤ 0.03) between disease and treatment states (Table 1).

**Figure 5:**
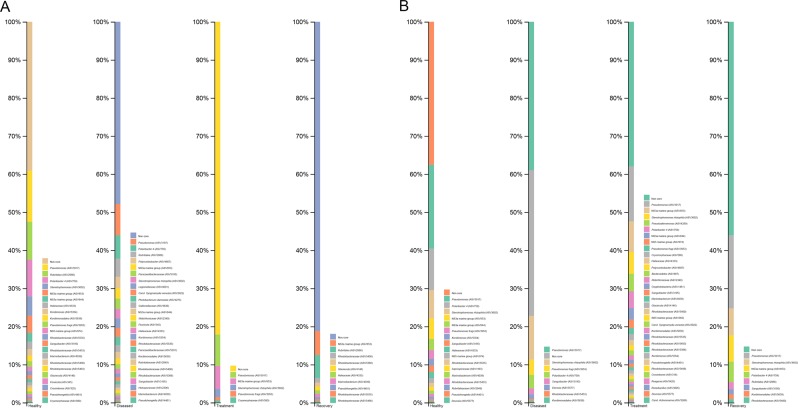
Core microbiota of seabass gill (**A**) and skin (**B**) at the ASV level. Distinctive bars represent relative abundance of each ASV for healthy, diseased, treatment and recovery states, labeled to the lowest taxonomic level possible.

Of the 55 ASVs recovered from the gill core microbiome across the four states, 21 were present in the healthy state, 26 in the diseased state, 5 in the treatment state and 10 in the recovery state (Fig. 5A). Four of these ASVs were unique to the healthy state, 11 unique to the diseased state and one to the recovery state (Fig. 5A). Of the 11 unique ASVs recovered from the gill core microbiome of diseased fish, one was identified as *Photobacterium damselae*. There were 8 other ASVs belonging to the *Photobacterium* genus, of which 7 were unique to the diseased state and one was found in all four states (Supplementary Table S1). This suggests that *P. damselae* is the most likely causative agent of the disease in the diseased fishes.

### Skin bacterial composition and diversity

Alpha-diversity estimates varied significantly across all states (RRPP, P ≤ 0.002; Table 1, Fig. 2B) and between states in the skin microbiome. They decreased significantly between healthy and diseased fish and increased significantly between diseased and treatment states (RRPP, P ≤ 0.003; Table 1, Fig. 2B). Beta-diversity estimates show significant differences across all states and between states (Adonis, P < 0.05; Table 1).

As for the skin microbiome, *Bacteroidetes, Proteobacteria* and *Verrucomicrobia* were the most abundant phyla retrieved across states, accounting for 87% to 93% of the sequences altogether (Table 2). The genera NS3a marine group, *Polaribacter 4*, *Pseudomonas* and *Stenotrophomonas* were the most abundant in all four states (Table 2, Supplementary Fig. S1). Moreover, *Pseudoalteromonas* accounted for 5% of the sequences in the treatment state, but only 0.1–1% in the remaining states (Table 2, Supplementary Fig. S1).

Mean proportions of the bacterial taxa varied significantly between states: (i) in healthy versus diseased states 4 taxa increased and 4 decreased; (ii) in diseased versus treatment states 5 taxa increased and 3 decreased; and (iii) in treatment versus recovery states 5 taxa increased, 2 decreased and 1 remained constant (Table 2, Fig. 4B). The 3 most abundant phyla and 5 most abundant genera all varied significantly (P ≤ 0.03) across the four states (Fig. 4B, Table 1). All taxa varied significantly between healthy and diseased states (P ≤ 0.01); all except one varied significantly between diseased and treatment states (P ≤ 0.02); and 2 genera varied significantly (P ≤ 0.02) between treatment and recovery states (Table 1).

A total of 43 ASVs formed the core microbiome of all four states, 17 were present in the healthy state, 8 were present in the diseased state, 33 in the treatment state and 8 in the recovery state (Fig. 5B). It is worth noticing that 2 ASVs were unique to the healthy state and 16 ASVs were unique to the treatment state.

## Discussion

In this study, we investigated the dynamics of the gill and skin microbiomes in 140 samples of the farmed seabass *Dicentrarchus labrax* during a natural disease outbreak and subsequent antibiotic treatment with oxytetracycline. We used high-throughput sequencing technology to generate 16S rRNA bacterial ASVs and examine changes in microbial composition and diversity over six weeks. We identified *Photobacterium damselae* as the most probable causative agent of disease.

The most abundant taxa found in the gill and skin microbiomes of healthy farmed seabass (*Dicentrarchus labrax*) belonged to the *Proteobacteria, Bacteroidetes* and *Verrucomicrobia* phyla. These phyla have been previously described as the most abundant in the gill and skin microbiomes of several teleosts^14,50–52^, including the seabass^16,53,54^. At the genus level, the most abundant taxa were the NS3a marine group*, Polaribacter 4, Pseudomonas*, and *Stenotrophomonas* in the gills and skin (Table 2, Supplementary Fig. S1, Fig. 4), and *Rubritalea* in the gills (Table 2, Supplementary Fig. S1, Fig. 4). These results are mainly in accordance with previously described microbiomes of healthy seabass^16,54^, including fish retrieved from the same farmed population during winter months^16^. However, one of the most abundant genera in the healthy seabass gill microbiome was *Polynucleobacter*^16^, which in the present study only accounted for 0.2% of the sequences in the healthy state, and 4–7% in the other three studied states (Table 2). Another compositional difference was the high abundance of *Stenotrophomonas* found in both tissues in apparently healthy individuals (Table 2) in this study, but not in Rosado *et al*.^16^. Several environmental factors known to impact microbiome composition, such as seasonality^55,56^ and water temperature^57^, could be driving these differences between our two studies.

The composition and diversity of the gill and skin seabass microbiomes varied differently during infection. Whereas in the skin there was a significant decrease in alpha-diversity between healthy and diseased fish, there were no significant differences in the gill microbiome. An overall decrease in microbial richness was also reported for the skin of Atlantic salmon as a result of infection with salmonid alphavirus^6^ and sea lice^15^; but interestingly, as in the present study, Legrand *et al*.^14^ reported significant differences in microbial richness between the skin of healthy and enteritis-infected yellowtail kingfish, but not in the gills.

Significant changes in beta-diversity occurred in both gills and skin, showing clear signs of dysbiosis in both tissues. In the skin microbiome of diseased fish, the abundance of taxa from the non-pathogenic NS3a marine group and *Polaribacter 4* decreased, whereas the pathogenic *Pseudomonas* and *Stenotrophomonas* significantly increased. *Pseudomonas* spp. almost doubled their abundance and largely dominated the skin microbiome of diseased fish. While the genus *Stenotrophomonas* contains important globally emergent fish pathogens (e.g. *Stenotrophomonas maltophilia*^58,59^), *Pseudomonas* harbors both opportunist fish pathogens (e.g. *P. baetica*, *P. chlororaphis*^60,61^; amongst others^8,62^) and taxa with known antimicrobial activity against fish pathogens (e.g. *Flavobacterium psychrophilum*^63^). For example, *P. fluorescens* is an important pathogen of carp and salmon^64,65^, but is also known to inhibit the growth of *Saprolegnia*, an oomycete that causes huge losses in aquaculture^66,67^. Importantly, a ten-fold increase of *Pseudoalteromonas*, which was not amongst the most abundant taxa in healthy fish, occurred in the skin of diseased fish. Species from this genus can inhibit the growth of both *Vibrio* spp. and *Photobacterium damselae*^6,68–71^, hence an increase of *Pseudoalteromonas* could lead to a decrease of the other two genera, as we have seen in the skin microbiomes of seabass transitioning from healthy to diseased states (from 2% to 0.7% and from 0.3% to 0.2%, respectively). In the gills of diseased fish, the majority of the most abundant bacterial genera in the healthy state (NS3a marine group, *Polaribacter* 4, *Pseudomonas*, *Rubritalea* and *Stenotrophomonas*) decreased significantly in abundance during infection, with the exception of *Polynucleobacter*. Amongst the most abundant taxa in the gill, only *Photobacterium* spp. was exclusively associated with diseased fish, where it showed a 25-fold increase. Similarly, all studies addressing the effects of parasitic infection on fish microbiomes reported significant changes in microbial composition^6,14,15^. Importantly, all of these studies reported an increase of potentially pathogenic taxa, which highlights the opportunistic nature of such pathogens^6,14,15^. Although *Photobacterium damselae* was only highly abundant in the diseased gill microbiome, the tissue with more significant shifts in overall bacterial composition (alpha-diversity) between healthy and diseased states was the skin. This is not totally unexpected, since it has been shown that this pathogen can unequally affect the microbiome of distinctive mucosal surfaces such as the skin and gill^14^.

The effects of the disease in the core microbiomes were also significant and again different between tissues, with a shift of core species in the gill and a decrease of core diversity in the skin from healthy to diseased states. A shift of the microbial assemblages with enrichment of specific groups was also described for the gill microbiome of the yellowtail killifish as a result of enteritis^14^.

Antibiotics administration can negatively impact host physiology in different ways (e.g., inhibiting mitochondrial gene expression^72^; decreasing enzymatic activity^46^), leading to dysbiosis and the emergence of antibiotic resistant bacteria^35,42–44,73^. Specifically, the reported effects of oxytetracycline in the gut microbiome of the Atlantic salmon showed a clear reduction in taxonomic diversity, becoming almost exclusively composed of the oxytetracycline resistant *Aeromonas* spp., which include the salmon pathogens *Aeromonas sobria* and *A. salmonicida*^74^. Similarly, in zebrafish, long-term exposure (6 weeks) to environmental concentrations of oxytetracycline, prompted both a decrease in gut microbial diversity and higher mortality when fish were challenged with the pathogen *A. hydrophila*^46^. The impact of broad-spectrum antibiotics in the skin microbiome of *Gambusia affinis* have also been assessed^41,42^. In this case, the use of rifampicin led to a decrease of diversity in the skin microbiome after 2.6 days of antibiotic administration. Additionally, as reported for zebrafish and Atlantic salmon, fish subjected to rifampicin antibiotic administration were more susceptible to infection due to osmotic stress and exhibited less growth compared to the control group, an effect that lasted one month after treatment^41,42^. A key difference with the present study is that the fish used by Carlson *et al*.^41,42^ were healthy before antibiotic administration. Importantly, our results showed that skin core diversity was higher in healthy than in recovery individuals, indicating a negative effect of disease and antibiotic use.

In the present study, administration of oxytetracycline resulted in a dramatic reduction of *Photobacterium* abundance in the gill microbiome, with this genus no longer being one of the most abundant taxa in the treatment and recovery states. This was expected given the reported sensitivity of *P. damselae* to several antibiotics, including oxytetracycline^38^. *Pseudoalteromonas*, however, remained one of the most abundant taxa in the skin microbiome during treatment perhaps due to the host innate immune response mediated by the skin microbiome, given the ability of this genus to produce antimicrobial metabolites that are correlated with host homeostasis^68^.

Previous studies on the impact of antibiotics on fish skin microbiomes showed that, even though stabilization of bacterial communities during recovery occurs, neither diversity nor composition returns to healthy-like values in the short term (after 1 week)^41,42^. Here the relative frequency of the most abundant taxa found in the skin microbiome of the seabass during the recovery period, which corresponded to 3 weeks, was similar to that in healthy individuals (P ≥ 0.1 for all taxa except the NS3a marine group). In the gill microbiome, however, differences in taxa proportions between the healthy and recovery states were significant for almost all of the most abundant taxa. Hence, although dysbiosis due to infection was more noticeable in the skin than in the gill, the microbial communities present in the skin seem to be more resilient than those of the gill. Importantly, although the abundance of *Photobacterium damselae* in the gill seemed to have been controlled through antibiotic administration, it increased significantly in the recovery state, surpassing its initial proportion in the healthy state.

In summary, the mucosal surfaces of fish, such as the gill and the skin, are constantly exposed to several pathogens in the aquatic environment and are crucial to prevent and/or control disease^1^. It has been shown that both infectious diseases and antibiotic treatment lead to a decrease in microbial diversity, which translates into a decrease in host immunity^14,42^. Here we described microbial changes in the gill and skin of adult seabass in response to a natural disease outbreak followed by a succeeding treatment with oxytetracycline. We showed that the gill and skin microbiomes are highly disturbed by both infection and antibiotic treatment, ultimately decreasing their diversity.

## Methods

### Ethical statement

This study monitored a natural infection and subsequent antibiotic treatment as part of routine procedures in a commercial fish farm. All animals were handled by the fish farm employees, our sampling through swabbing was non-invasive and fish were released unharmed with no mortalities observed. According to the Portuguese legislation DL N° 113/2013, our work does not involve animal experimentation and therefore is exempted from the need of ethical approval.

### Experimental design, sample collection and preparation

Ten individuals of seabass were collected once a week between August 21 and October 3, 2016, from the same rearing tank in a commercial fish farm located in the estuarine environment of the Ria Formosa (Portimão), southern Portugal. Fish were hatched at September 26, 2014 and entered the growth facility at March 6, 2015. Fish were kept in an open water circulation system in a semi-intensive farming facility, where water is supplied to each tank from the estuary. Fish were kept at a density of ca. 3 kg/m^3^ corresponding to roughly 100 fish/tank with fish weighting on average 281 g. Given that it was not possible to tag individual fish and the unlikelihood of re-sampling the same individuals every week, a subset of samples believed to be representative of the population was chosen, i.e. 10 individuals (~ 10%), and for statistical purposes individuals were considered as pseudo-replicates. All fish were fed with the same commercial feed and they shared the same clinical history. Individuals were randomly caught using a fishing pole and skin and gill swabs were collected immediately using tubed sterile dry swabs (Medical Wire & Equipment, UK). Skin samples were taken by swabbing several times along the right upper lateral part of the fish from head to tail, while gill swabs were taken from the right filaments between the first and second arch. Due to the non-invasive nature of our sampling procedure, it was not possible to ascertain the sex of the individuals sampled; however, we do not expect this to impact our conclusions since, to the best of our knowledge, no gender bias in microbiome composition has ever been reported for skin or gill of piscine hosts. Swabs were immediately stored at −20 °C until transported on dry ice to the CIBIO-InBIO laboratory by airmail where they were kept at −80 °C until further processing.

To assess gill and skin microbiome dynamics in the seabass during a disease outbreak and under oxytetracycline treatment after infection, fish were sampled in 4 different states: healthy, diseased, treatment and recovery (Fig. 1). During the healthy state (August 21 and 29), all fish specimens were considered healthy due to a lack of visible disease symptoms, such as external lesions or behavioural alterations. On September 8 fish began to die in the farming tanks, showing symptoms of disease, and treatment with oxytetracycline antibiotic (a broad-spectrum tetracycline) was initiated, being administrated at 35 g/Kg through commercial feed for at lasted 8 days. On the same day, smears from spleen and kidney were collected for culture using Bionor kits DE020, MONO-VA-50 for *Vibrio anguillarum* and DL020, MONO-Pp-50 for *Photobacterium piscicida*. Agglutination essays were not conclusive and, at that stage, the causative agent of the disease was unknown. We do not have samples from September 8, hence we used the samples from our closest time point, September 5, which we classified as potentially diseased (i.e., diseased state). Antibiotic treatment lasted until September 16 and fish were sampled on September 12; this sample point corresponded to the treatment state. Then, three additional time points were sampled (September 19 and 26, and October 3), when fish were no longer dying or presented signs of infection; these three time points corresponded to the recovery state.

Total DNA from 140 fish samples (70 skin and 70 gills) was extracted using the PowerSoil DNA Isolation Kit (QIAGEN, Netherlands), following the manufacturer’s protocol. DNA extractions were shipped in dry ice to the University of Michigan Medical School (USA) for amplification and sequencing on a single run of the Illumina MiSeq platform according to the protocol of Kozich *et al*.^75^ Each sample was amplified for the V4 (~250 bp) hypervariable region of the 16S rRNA gene, using the primers in Caporaso *et al*.^76^ This region has been widely used to characterize microbiomes from vertebrates (Earth Microbiome Project^77^), including fish^42,78–80^.

### Data and statistical analysis

Raw FASTQ files were analyzed using the Quantitative Insights Into Microbial Ecology 2 (QIIME2; release 2018.4) platform. Clean sequences were aligned against the SILVA (132 release) reference database^81^ using the DADA2 pipeline^82^. A feature table containing amplicon sequence variants (ASVs) was constructed and normalized using the negative binomial distribution^83^. The core microbiome was assessed at the ASV level for the gill and skin of seabass for each state (healthy, diseased, treatment and recovery) separately. An ASV was considered as part of the core microbiome if present in 100% of the samples from each state. Core diversity is here defined as number of ASVs represented in a given group.

Microbial alpha-diversity (intra-sample) was calculated using Shannon, ACE, Fisher and Faith’s phylogenetic diversity (PD) indices as implemented in the R package phyloseq^84^. Microbial beta-diversity (inter-sample) was estimated using phylogenetic Unifrac (unweighted and weighted) and Bray-Curtis distances. Dissimilarity between samples was assessed by principal coordinates analysis (PCoA). Variation in microbial alpha-diversity and the mean proportions of the most abundant taxa (with more than 4% of all reads) were assessed using linear models with randomized residuals in a permutation procedure (RRPP). Differences in community composition (beta-diversity) were tested using permutational multivariate analysis of variance (PERMANOVA) with 1,000 permutations as implemented in the *adonis* function of the R vegan package^85^. All statistical analyses were carried out separately for the gills and skin. All statistical analyses were performed in R-studio v1.0.143^86^.

## Supplementary information

Table S1

Figure S1

## Data Availability

The raw sequences are available at NCBI Sequence Read Archive (SRA) database within the BioProject ID PRJNA575053.

## References

[1] Trivedi B (2012). Microbiome: the surface brigade. Nature..

[2] Gómez GD, Balcázar JL (2007). A review on the interactions between gut microbiota and innate immunity of fish. FEMS Immunol. Med. Microbiol..

[3] Kelly C, Salinas I (2017). Under pressure: interactions between commensal microbiota and the teleost immune system. Front. Immunol..

[4] Lee YK, Mazmanian SK (2010). Has the microbiota played a critical role in the evolution of the adaptive immune system?. Science.

[5] Mohammed HH, Arias CR (2015). Potassium permanganate elicits a shift of the external fish microbiome and increases host susceptibility to columnaris disease. Vet. Res..

[6] Reid KM (2017). Salmonid alphavirus infection causes skin dysbiosis in Atlantic salmon (*Salmo salar* L.) post-smolts. PloS One..

[7] Larsen A, Tao Z, Bullard SA, Arias CR (2013). Diversity of the skin microbiota of fishes: evidence for host species specificity. FEMS Microbiol. Ecol..

[8] Boutin S, Bernatchez L, Audet C, Derôme N (2013). Network analysis highlights complex interactions between pathogen, host and commensal microbiota. PLoS One..

[9] Zha Y, Eiler A, Johansson F, Svanbäck R (2018). Effects of predation stress and food ration on perch gut microbiota. Microbiome.

[10] Chiarello M (2018). Skin microbiome of coral reef fish is highly variable and driven by host phylogeny and diet. Microbiome.

[11] Galbraith H (2018). Exposure to synthetic hydraulic fracturing waste influences the mucosal bacterial community structure of the brook trout (*Salvelinus fontinalis*) epidermis. AIMS Microbiol..

[12] Ye L, Amberg J, Chapman D, Gaikowski M, Liu WT (2014). Fish gut microbiota analysis differentiates physiology and behavior of invasive Asian carp and indigenous American fish. ISME J..

[13] Pratte ZA, Besson M, Hollman RD, Stewart FJ (2018). The gills of reef fish support a distinct microbiome influenced by host-specific factors. Appl. Environ. Microbiol..

[14] Legrand TP (2018). The inner workings of the outer surface: skin and gill microbiota as indicators of changing gut health in yellowtail kingfish. Front. Microbiol..

[15] Llewellyn MS (2017). Parasitism perturbs the mucosal microbiome of Atlantic salmon. Sci. Rep..

[16] Rosado D, Pérez-Losada M, Severino R, Cable J, Xavier R (2019). Characterization of the skin and gill microbiomes of the farmed seabass (*Dicentrarchus labrax*) and seabream (*Sparus aurata*). Aquaculture.

[17] Givens CE, Ransom B, Bano N, Hollibaugh JT (2015). Comparison of the gut microbiomes of 12 bony fish and 3 shark species. Mar. Ecol. Prog. Ser..

[18] Li E (2018). Gut microbiota and its modulation for healthy farming of Pacific white shrimp *Litopenaeus vannamei*. Rev. Fish. Sci. Aquac..

[19] Califano G (2017). Molecular taxonomic profiling of bacterial communities in a gilthead seabream (*Sparus aurata*) hatchery. Front. Microbiol..

[20] Rud I (2017). Deep sequencing of the bacterial microbiota in commercial-scale recirculating and semiclosed aquaculture systems for Atlantic salmon post-smolt production. Aquac. Eng..

[21] Hess S, Wenger AS, Ainsworth TD, Rummer JL (2015). Exposure of clownfish larvae to suspended sediment levels found on the Great Barrier Reef: impacts on gill structure and microbiome. Sci. Rep..

[22] Llewellyn MS, Boutin S, Hoseinifar SH, Derome N (2014). Teleost microbiomes: the state of the art in their characterization, manipulation and importance in aquaculture and fisheries. Front. Microbiol..

[23] FAO Aquaculture Department: Statistics and Information Service FishStatJ: Universal software for fishery statistical time series (2011).

[24] Toranzo AE, Magarinos B, Romalde JL (2005). A review of the main bacterial fish diseases in mariculture systems. Aquaculture..

[25] Faílde LD (2014). Immunohistochemical diagnosis of tenacibaculosis in paraffin‐embedded tissues of Senegalese sole *Solea senegalensis* Kaup, 1858. J. Fish Dis..

[26] Zlotkin A, Hershko H, Eldar A (1998). Possible transmission of *Streptococcus iniae* from wild fish to cultured marine fish. Appl. Environ. Microbiol..

[27] Romalde JL (2008). *Streptococcus phocae*, an emerging pathogen for salmonid culture. Vet. Microbiol..

[28] Rivas AJ, Balado M, Lemos ML, Osorio CR (2011). The *Photobacterium damselae* subsp. *damselae* hemolysins damselysin and HlyA are encoded within a new virulence plasmid. Infect. Immun..

[29] Tao Z (2018). An outbreak of *Photobacterium damselae* subsp. *damselae* infection in cultured silver pomfret *Pampus argenteus* in Eastern China. Aquaculture.

[30] Terceti MS, Ogut H, Osorio CR (2016). *Photobacterium damselae* subsp*. damselae*, an emerging fish pathogen in the Black Sea: evidences of a multiclonal origin. Appl. Environ. Microbiol..

[31] Terceti MS (2018). Molecular epidemiology of *Photobacterium damselae s*ubsp. *damselae* outbreaks in marine rainbow trout farms reveals extensive horizontal gene transfer and high genetic diversity. Front. Microbiol..

[32] Labella, A., Berbel, C., Manchado, M., Castro, D. & Borrego, J. J. *Photobacterium damselae* subsp. *damselae*, an emerging pathogen affecting new cultured marine fish species in southern Spain. In *Recent advances in fish farms*. InTech (2011).

[33] Pedersen K, Skall HF, Lassen‐Nielsen AM, Bjerrum L, Olesen NJ (2009). *Photobacterium damselae* subsp*. damselae*, an emerging pathogen in Danish rainbow trout, *Oncorhynchus mykiss* (Walbaum), mariculture. J. Fish Dis..

[34] Rivas AJ, Lemos ML, Osorio CR (2013). *Photobacterium damselae* subsp. *damselae*, a bacterium pathogenic for marine animals and humans. Front. Microbiol..

[35] Essam HM, Abdellrazeq GS, Tayel SI, Torky HA, Fadel AH (2016). Pathogenesis of *Photobacterium damselae* subspecies infections in sea bass and sea bream. Micro. Pathogenesis..

[36] Bakopoulos V (2003). Vaccination trials of sea bass, *Dicentrarchus labrax* (L.), against *Photobacterium damsela subsp. piscicida*, using novel vaccine mixtures. J. Fish Dis..

[37] Byadgi O (2018). Immunogenicity of inactivated formalin-killed *Photobacterium damselae* subsp. *piscicida* combined with Toll-like receptor 9 agonist in *Cobia rachycentron canadum*. Aquaculture..

[38] Abdel-Aziz M, Eissa AE, Hanna M, Okada MA (2013). Identifying some pathogenic *Vibrio/Photobacterium* species during mass mortalities of cultured Gilthead seabream (*Sparus aurata*) and European seabass (*Dicentrarchus labrax*) from some Egyptian coastal provinces. Int. J. Vet. Sci. Med..

[39] Rodrigues S, Antunes SC, Correia AT, Nunes B (2017). Rainbow trout (*Oncorhynchus mykiss*) pro-oxidant and genotoxic responses following acute and chronic exposure to the antibiotic oxytetracycline. Ecotoxicology.

[40] Yonar ME, Yonar SM, Silici S (2011). Protective effect of propolis against oxidative stress and immunosuppression induced by oxytetracycline in rainbow trout (*Oncorhynchus mykiss*, W.). Fish Shellfish Immunol..

[41] Carlson JM, Hyde ER, Petrosino JF, Manage AB, Primm TP (2015). The host effects of *Gambusia affinis* with an antibiotic-disrupted microbiome. Comp. Biochem. Phys. C..

[42] Carlson JM, Leonard AB, Hyde ER, Petrosino JF, Primm TP (2017). Microbiome disruption and recovery in the fish *Gambusia affinis* following exposure to broad-spectrum antibiotic. Infect. Drug Resist..

[43] Pindling S, Azulai D, Zheng B, Dahan D, Perron GG (2018). Dysbiosis and early mortality in zebrafish larvae exposed to subclinical concentrations of streptomycin. FEMS Microbiol. Lett..

[44] Liu Y (2012). Gibel carp *Carassius auratus* gut microbiota after oral administration of trimethoprim/sulfamethoxazole. Dis. Aquat. Organ..

[45] Narrowe AB (2015). Perturbation and restoration of the fathead minnow gut microbiome after low-level triclosan exposure. Microbiome..

[46] Zhou L (2018). Environmental concentrations of antibiotics impair zebrafish gut health. Environ. Pollut..

[47] Chiu TH, Kao LY, Chen ML (2013). Antibiotic resistance and molecular typing of *Photobacterium damselae* subsp. *damselae*, isolated from seafood. J. Appl. Microbiol..

[48] Nonaka L (2012). Novel conjugative transferable multiple drug resistance plasmid pAQU1 from *Photobacterium damselae* subsp. *damselae* isolated from marine aquaculture environment. Microbes Environ..

[49] Rigos G, Troisi GM (2005). Antibacterial agents in Mediterranean finfish farming: a synopsis of drug pharmacokinetics in important euryhaline fish species and possible environmental implications. Revi. Fish Biol. Fisher..

[50] Boutin S, Sauvage C, Bernatchez L, Audet C, Derôme N (2014). Inter individual variations of the fish skin microbiota: host genetics basis of mutualism?. PLoS One..

[51] Lowrey, L., Woodhams, D. C., Tacchi, L. & Salinas, I. Topographical mapping of the rainbow trout (*Oncorhynchus mykiss*) microbiome reveals a diverse bacterial community in the skin with antifungal properties. *Appl. Environ. Microb*. AEM-01826 (2015). 10.1128/AEM.01826-15 PMC4561705 26209676

[52] Tapia-Paniagua ST, Ceballos-Francisco D, Balebona MC, Esteban MÁ, Moriñigo MÁ (2018). Mucus glycosylation, immunity and bacterial microbiota associated to the skin of experimentally ulcered gilthead seabream (*Sparus aurata*). Fish Shellfish Immunol..

[53] Chiarello, M., Villéger, S., Bouvier, C., Bettarel, Y. & Bouvier, T. High diversity of skin-associated bacterial communities of marine fishes is promoted by their high variability among body parts, individuals and species. *FEMS Microbiol. Ecol*. **91** (2015). 10.1093/femsec/fiv061 26048284

[54] Pimentel T, Marcelino J, Ricardo F, Soares AM, Calado R (2017). Bacterial communities 16S rDNA fingerprinting as a potential tracing tool for cultured seabass *Dicentrarchus labrax*. Sci. Rep..

[55] Larsen AM, Bullard SA, Womble M, Arias CR (2015). Community structure of skin microbiome of gulf killifish, *Fundulus grandis*, is driven by seasonality and not exposure to oiled sediments in a Louisiana salt marsh. Microb. Ecol..

[56] de Bruijn I, Liu Y, Wiegertjes GF, Raaijmakers JM (2017). Exploring fish microbial communities to mitigate emerging diseases in aquaculture. FEMS Microbiol. Ecol..

[57] Lokesh J, Kiron V (2016). Transition from freshwater to seawater reshapes the skin associated microbiota of Atlantic salmon. Sci. Rep..

[58] Brooke JS (2012). *Stenotrophomonas maltophilia*: an emerging global opportunistic pathogen. Clin. Microbiol. Rev..

[59] Abraham, T. J. & Adikesavalu, H. Association of *Stenotrophomonas maltophilia* in African catfish, *Clarias gariepinus* (Burchell, 1822) fry mortalities with dropsy. *Int. J. Aquac*. **6** (2016).

[60] López JR (2012). *Pseudomonas baetica* sp. nov., a fish pathogen isolated from wedge sole, *Dicologlossa cuneata* (Moreau). Int. J. Syst. Evol. Microbiol..

[61] Hatai, K., Egusa, S., Nakajima, M. & Chikahata, H. *Pseudomonas chlororaphis* as a fish pathogen. *B. Jpn. Soc. Sci. Fish*. **41** (1975).

[62] Pridgeon JW, Klesius PH (2012). Major bacterial diseases in aquaculture and their vaccine development. Anim. Sci. Rev..

[63] Korkea‐Aho TL, Heikkinen J, Thompson KD, Von Wright A, Austin B (2011). *Pseudomonas* sp. M174 inhibits the fish pathogen *Flavobacterium psychrophilum*. J. Appl. Microbiol..

[64] Austin, B. & Austin, D. A. Characteristics of the diseases. In *Bacterial Fish Pathogens: Diseases of Farmed and Wild Fish*15–46 (Springer, 2012).

[65] Loch TP, Scribner K, Tempelman R, Whelan G, Faisal M (2012). Bacterial infections of Chinook salmon, *Oncorhynchus tshawytscha* (Walbaum), returning to gamete collecting weirs in Michigan. J. Fish Dis..

[66] Liu Y (2015). Diversity of aquatic *Pseudomonas* species and their activity against the fish pathogenic oomycete *Saprolegnia*. PloS One..

[67] van West P (2006). *Saprolegnia parasitica*, an oomycete pathogen with a fishy appetite: new challenges for an old problem. Mycologist.

[68] Offret C (2016). Spotlight on antimicrobial metabolites from the marine bacteria *Pseudoalteromonas*: chemodiversity and ecological significance. Mar. Drugs..

[69] Richards, G. P. *et al*. Mechanisms for *Pseudoalteromonas piscicida*-induced killing of vibrios and other bacterial pathogens. *Appl. Environ. Microbiol*. AEM-00175 (2017). 10.1128/AEM.00175-17 PMC5440704 28363962

[70] Lloyd MM, Pespeni MH (2018). Microbiome shifts with onset and progression of Sea Star Wasting Disease revealed through time course sampling. Sci. Rep..

[71] Papaleo MC (2012). Sponge-associated microbial Antarctic communities exhibiting antimicrobial activity against *Burkholderia cepacia* complex bacteria. Biotechnol. Adv..

[72] Morgun A (2015). Uncovering effects of antibiotics on the host and microbiota using transkingdom gene networks. Gut.

[73] Gaulke, C. A., Martins, M. L., Watral, V., Kent, M. L. & Sharpton, T. J. Parasitic Infection by *Pseudocapillaria tomentosa* is associated with a longitudinal restructuring of the Zebrafish gut microbiome. *bioRxiv*, p.076596 (2016).

[74] Navarrete P, Mardones P, Opazo R, Espejo R, Romero J (2008). Oxytetracycline treatment reduces bacterial diversity of intestinal microbiota of Atlantic salmon. J. Aquat. Anim. Health..

[75] Kozich JJ, Westcott SL, Baxter NT, Highlander SK, Schloss PD (2013). Development of a dual-index sequencing strategy and curation pipeline for analyzing amplicon sequence data on the MiSeq Illumina sequencing platform. Appl. Environ. Microbiol..

[76] Caporaso JG (2011). Global patterns of 16S rRNA diversity at a depth of millions of sequences per sample. Proc. Natl. Acad. Sci..

[77] Gilbert JA, Jansson JK, Knight R (2014). The Earth Microbiome project: successes and aspirations. BMC Biol..

[78] Llewellyn MS (2015). The biogeography of the Atlantic salmon (*Salmo salar*) gut microbiome. ISME J..

[79] Nielsen S, Walburn JW, Vergés A, Thomas T, Egan S (2017). Microbiome patterns across the gastrointestinal tract of the rabbitfish *Siganus fuscescens*. Peer J..

[80] Wang J (2017). Effects of fish meal replacement by soybean meal with supplementation of functional compound additives on intestinal morphology and microbiome of Japanese seabass (*Lateolabrax japonicus*). Aquac. Res..

[81] Quast C (2012). The SILVA ribosomal RNA gene database project: improved data processing and web-based tools. Nucleic Acids Res..

[82] Callahan BJ (2016). DADA2: high-resolution sample inference from Illumina amplicon data. Nat. Methods..

[83] McMurdie PJ, Holmes S (2014). Waste not, want not: why rarefying microbiome data is inadmissible. PLoS Comput. Biol..

[84] McMurdie PJ, Holmes S (2013). Phyloseq: an R package for reproducible interactive analysis and graphics of microbiome census data. PLoS One..

[85] Oksanen, J. *et al*. The vegan package. *Community Ecology Package*, http://r-forge.r-project.org/projects/vegan (2008).

[86] Studio, R. RStudio: Integrated Development Environment for R. RStudio Inc, Boston, Massachusetts (2012).

