# Supplementary material for: Effects of disease, antibiotic treatment and recovery trajectory on the microbiome of farmed seabass (Dicentrarchus labrax)
Source: Sci Rep. 2019 Dec 12;9:18946. doi: 10.1038/s41598-019-55314-4 (PMC6908611; doi:10.1038/s41598-019-55314-4)
Supplement: Supplementary file 2 — Figure S1 [file 41598_2019_55314_MOESM2_ESM.pdf]

Supplementary information

Effects of disease, antibiotic treatment and recovery trajectory on the microbiome of farmed seabass (*Dicentrarchus labrax*)

Daniela Rosado, Raquel Xavier*, Ricardo Severino, Fernando Tavares, Jo Cable, Marcos Pérez-Losada

*Corresponding author: Raquel Xavier - raq.xavier@cibio.up.pt

Supplementary Figure

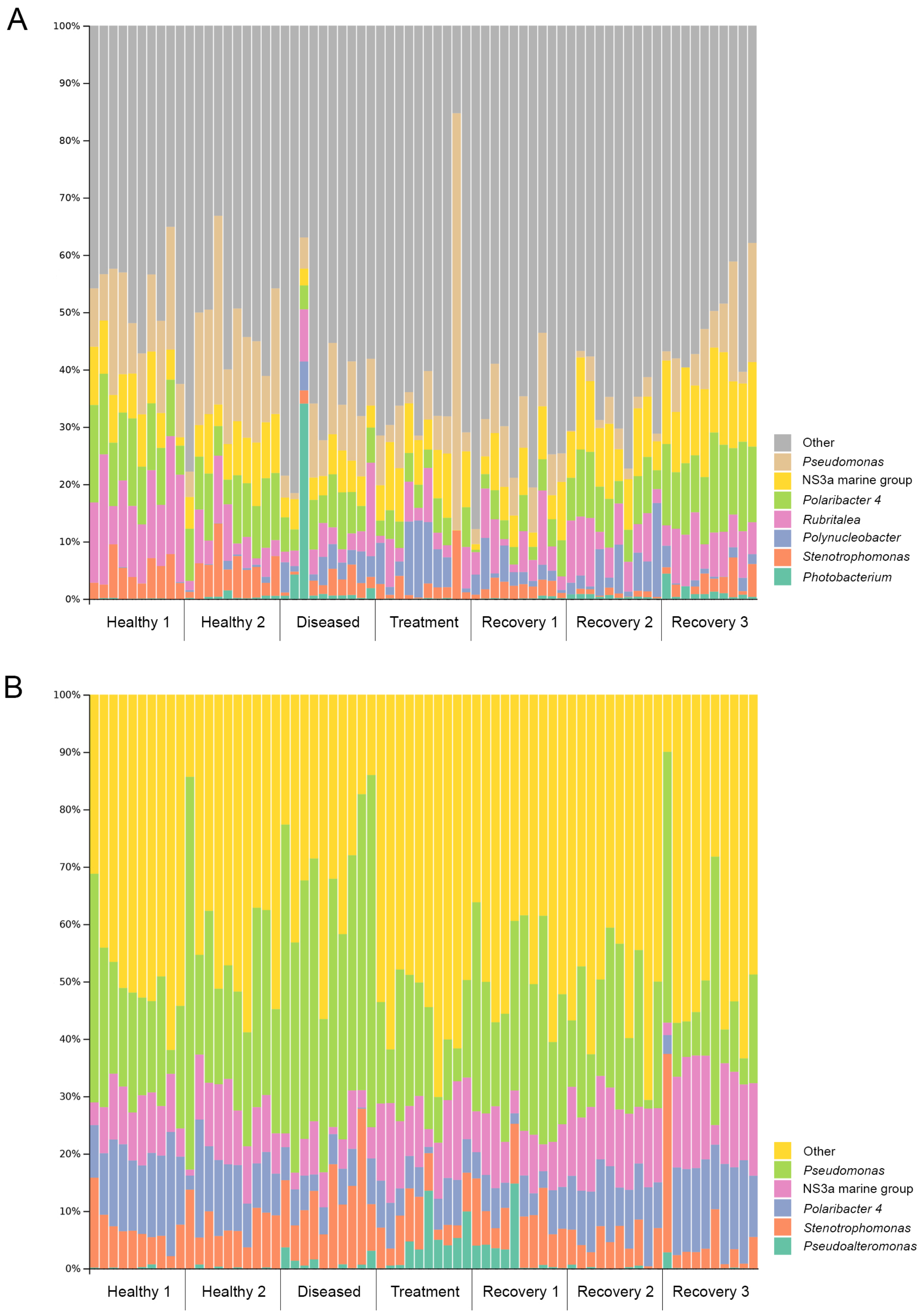

Supplementary Figure S1: Individual microbial variation of the most abundant (>5%) genera recovered from the gill (A) and skin (B) of the seabass. Distinctive bars represent relative abundance of each genus.
